# Whole Time Series Data Streams Clustering: Dynamic Profiling of the Electricity Consumption

**DOI:** 10.3390/e22121414

**Published:** 2020-12-15

**Authors:** Krzysztof Gajowniczek, Marcin Bator, Tomasz Ząbkowski

**Affiliations:** Department of Artificial Intelligence, Institute of Information Technology, Warsaw University of Life Sciences-SGGW, 02-776 Warsaw, Poland; marcin_bator@sggw.edu.pl (M.B.); tomasz_zabkowski@sggw.edu.pl (T.Z.)

**Keywords:** clustering, data stream, machine learning, smart metering, time series

## Abstract

Data from smart grids are challenging to analyze due to their very large size, high dimensionality, skewness, sparsity, and number of seasonal fluctuations, including daily and weekly effects. With the data arriving in a sequential form the underlying distribution is subject to changes over the time intervals. Time series data streams have their own specifics in terms of the data processing and data analysis because, usually, it is not possible to process the whole data in memory as the large data volumes are generated fast so the processing and the analysis should be done incrementally using sliding windows. Despite the proposal of many clustering techniques applicable for grouping the observations of a single data stream, only a few of them are focused on splitting the whole data streams into the clusters. In this article we aim to explore individual characteristics of electricity usage and recommend the most suitable tariff to the customer so they can benefit from lower prices. This work investigates various algorithms (and their improvements) what allows us to formulate the clusters, in real time, based on smart meter data.

## 1. Introduction

The advances in smart metering solutions have enabled that gathering information about customer power consumption in real time is feasible and it can be successfully used for data exploration to bring actionable recommendations. The data (in the form of a time series) from the smart grid still makes challenges to analyze it due to the very large size, high dimensionality, skewness, sparsity, and number of seasonal fluctuations, including daily and weekly effects. Although the analysis requires a lot of effort to discover the segmentation of entities based on their electricity consumption data, the benefits, as the result of the data insights, would be very appealing to the electricity providers [1]. By supplying providers with demand response predictions on aggregated level, due to segmentation (other terms such as clustering and grouping are used interchangeably), and revealing the real economic structure of the entities (e.g., individual users, households, small business) the goal is to fit into the integrated planning system, where the appropriate real-time actions could be proposed to meet the system demands effectively [2]. Well recognized consumption patterns itself are also a source of valuable insight to determine optimal tariff rates for the users and to deal with the spikes in electricity demand.

The analysis of the data streams (in this article we deal with time series and therefore we will use term time series data streams as well) coming from the grid over consecutive time windows allows for a better understanding of the usage characteristics. With the data arriving in a sequential form the underlying distribution is subject to changes over the time intervals what is referred to as concept drift [3,4]. For example, the changes in smart meter streaming data may be the result of many factors, including those related to weather conditions, to week days or those related to price incentives [5]. It is often observed that smart meter readings received at an instant intervals may have a dynamic distribution or may contain a large number of sparse and missing values. Therefore, traditional algorithms are not applicable directly nor suitable for these type of data as they extract patterns from data by assuming the global properties (what requires the complete training data set), rather than capturing the local ones.

Time series data streams have their own specifics in terms of data exploration and processing, because, usually, it is not possible to process the whole history in memory. The reason for that is that data are coming very fast so the processing and the analysis should be done incrementally using sliding windows (overlapping or non-overlapping) or using other approaches like the stochastic learning weak estimators [6]. Classical clustering algorithms aim to divide a set of objects (observations) into groups so that objects in the same group are more similar to each other than objects in other groups. The literature on time series data stream clustering makes a distinction in terms of what is the subject of grouping [3]. The first approach tries to cluster observations from a single univariate or multivariate time series data stream through lots of promising tools and methods [7]. On the other hand, second approach tries to analyze multiple time series data streams, generated by several sources (e.g., smart meters), in order to find a division of sources. In literature the latter problem is also known as attribute clustering [8]. Despite the proposal of many clustering techniques dedicated for the first approach, only a few of them are dedicated to the second approach. Due to that in this article we focus on multiple time series data streams clustering, as this is one of the most important challenge in data stream mining.

In many countries, all over the world, the retail electricity demand side of the market consists of several groups of end users. In Poland, for instance, the vast majority of consumers belong to the so-called tariff group G (mostly households). Other end users belong to so-called tariff groups A (top, strategic customers), B (large, key customers) which are supplied from the high and medium voltage grid, while group C consists of customers connected to the low voltage grid, consuming electricity for business purposes and they are called commercial customers [2]. For low-voltage households, operators have set up several different tariff groups which differ in the time zone (single or two time zone meters) and whether or not electricity is used for heating. The most general tariff group for households is G11, i.e., customers with single time zone meters and flat price per kWh. The other tariff groups, G12, G12r, and G12w, are time and weekdays. G12 is effective between 10 p.m. and 6 a.m. and between 1 p.m. and 3 p.m., while G12w is additionally effective during the weekends (between 10 p.m. on Friday and 7 a.m. on Monday). G12r is effective seven days a week between 10 p.m. and 7 a.m. and between 1 p.m. and 4 p.m. 

The main goal of this article is to investigate technical aspects of the existing clustering algorithms for time series data streams. The secondary goal is to explore individual characteristics of electricity usage and to recommend the most suitable tariff to the customers so they can benefit from lower prices, thus optimize the expenses. The research shall be conducted on the basis of a dataset provided by the Irish Commission for Energy Regulation (CER; detailed analysis) [5] and two other datasets, which are described later. We investigate various algorithms (and their improvements) what allows us to formulate the clusters in real time based on smart meter data. Basically, we develop a clustering approach applicable for data streams with the primary motivation to create well defined user profiles what may further allow to create more predictable groups of customers. The contribution of this article can be summarized as follows:

- We have created the framework and measures to compare and to evaluate time series data streams clustering algorithms;
- New Fast Fourier Transformation based features were created (calculated in liner time) to compress and to represent time series using the business context;
- Comparative study between the state-of-the-art time series data streams clustering algorithms was prepared;
- Comparative study between overlapping and non-overlapping windows and their impact on the choice of an optimal tariff was prepared; and
- Finally, an approach for dynamic consumer segmentation and prediction of an optimal tariff was proposed.

We believe that our contribution would address the gap related to those aspects of dynamic profiling where there was no clear conclusion with regards to the benefit of using overlapping vs. non-overlapping windows and the impact of those on the results of clustering algorithms.

The remainder of this paper is organized as follows: Section 2 provides an overview of the similar research problems for data stream time series clustering and electricity consumption segmentation. In Section 3, the theoretical framework of the proposed algorithm is presented. In Section 4, the research framework is outlined, including the details of numerical implementation, evaluation measure description, and algorithm parameter settings. Section 5 outlines the experiments and presents the discussion of the results. The paper ends with concluding remarks in Section 6.

## 2. Literature Review

Whilst the vast majority of customers belong to a single tariff with high volatility within the group, it creates a number of challenges, including short-term and long-term forecasting to meet the demand side response (DSR) of electricity operators, not to mention the stability of the whole network [9]. Obviously, daily energy consumption does not depend only on the composition of the customer’s tariffs, but also it depends on many external factors related to specific days, atmospheric phenomena, and weather conditions [10]. Due to that, there is a need for an objective approach to increase the effectiveness and efficiency of network management and operations by dividing mass markets into consumer groups with clearly similar patterns of behavior. This can be supported by statistical clustering methods what helps to formulate valid and meaningful clusters based on the available measurements data e.g., hourly.

Given the huge number of low-voltage customers, especially households, hourly measuring and recording equipment are a serious shortage. Both, the future demand and the initial settlement of customers are determined based on the load shape associated with specific tariff group. In that case, a similar energy demand structure determines the number of groups. Statistical and engineering techniques [11,12,13,14], time series [15,16,17], and neural networks [16,18,19] are used for load profiling. Based on the literature review, there is a clear and increasingly recognizable research trend that addresses the challenges of segmentation of electricity end-users. For example, the application of the k-means algorithm for clustering of the daily load profiles of individual users was described in [17,20,21,22]. A comparison of clustering algorithms for classifying household electricity consumers Kohonen’s self-organization map (SOM), and including hierarchical clustering, was analyzed by [2,23].

The literature on data streams clustering is quite extensive and includes the methods (1) aiming at grouping of the observations of a single data stream; and (2) proposals that monitor the proximity between multiple data streams in order to find the division of streams into clusters. The state-of-the-art survey of a multivariate or single univariate data stream clustering methods is available in [3]. Authors have presented a comprehensive survey on this phenomenon which discusses various types of data stream clustering techniques and the corresponding challenges. So far, most of the attention has been devoted to observations-based data streams clustering, which focuses on clustering of the observations from the single data stream. Reference is made to several categories of methods, including: Grid-based stream methods, partitioning stream methods, density-based stream methods, hierarchical stream methods, and growing neural gas-based methods. The flagship methods in those categories are: Str-FSFDP [24], MuDi [25], D-Stream [26], ClusStream [27], DBSTREAM [28], BIRCH [29], E-Stream [30], and StreamKM++ [31]. 

A more detailed analysis of the literature on grouping of multiple data streams (or time series stream), which is the subject of this article, is desired. For example, the recent methods are constructed in a way to ensure the division of streams over time [32,33,34,35,36,37,38,39]. All of them monitor the proximity of data streams using a record flow and introduce some strategies to obtain partitioning of streams into a set of clusters. Other interesting methods, such as [40,41,42,43], are focused on monitoring proximity between streams, but these do not include a grouping stage.

In the broader context of the techniques used for electricity consumption data driven by explosive growth of time-series data and the capability of the methods there are interesting attempts which propose a cohort of dominant data set selection algorithms for electricity consumption time series with a focus on discriminating the dominant data set that is a small data set but capable of representing the key information carried by time series with an arbitrarily small error rate [44].

Authors in [34] discussed the clustering on-demand framework (COD) involving a single data scan to derive online statistics. The COD consists of two stages, namely the online maintenance (providing an effective mechanism for maintaining hierarchical summaries of data streams) and offline clustering (finding approximations of desired sub-streams from the summary hierarchy according to cluster queries). Based on this algorithm Chen [39] introduced the CORREL-cluster algorithm offering a time horizon segmentation scheme and statistical information storage for each time segment. 

A tree-like hierarchy of clusters evolving with the data and using a top-down strategy has been introduced in [38]. The Online Divisive-Agglomerative Clustering algorithm (ODAC), incorporates correlation-based measure of similarity between time series, dividing each node by the furthest pair of streams. Due to the splitting and merging, operators algorithm is able to detect and to adapt to the data in the presence of the concept drift. The performance of the ODAC algorithm has been next improved by TS-Stream algorithm which calculates several descriptive time series measures and builds a decision tree [37]. Adequate measures are selected on the basis of the criterion of minimizing variance. As previously, the algorithm can gradually expand or reduce the tree according to changes in the stream that change the node variance. Finally, in [45] authors have presented an extended version of the TS-Stream algorithm, that overcomes some base algorithm drawbacks. After those modifications the final tree structure reaches its full size immediately and it can have leaves with the number of time series above a certain threshold (otherwise the tree would be very complex and deep).

Algorithm called IDEStream has been introduced by [39]. In this approach an autoregressive modelling (AR) is used to measure the correlation between data streams and it uses the estimated frequency spectrum to extract the relevant data stream characteristics such as attenuation rate, phase, and amplitude. Authors in [36] presented a two phase algorithm which uses a gamma mixture model to identify dense units of incoming data in the first phase. Aim of the second phase is to cluster the time series from one time window, while third phase performs incremental clustering between received groups of two consecutive time windows.

In [32] authors have developed a powerful online version of the fuzzy C-means algorithm (FCM-DS), allowing to quickly calculate the approximate distance between the streams, thanks to the scalable online transformation of the original data. In [35] authors have presented an algorithm called ClipStream where time-series are compressed and represented by interpretable features separated from clipped representation. Next, based on such data transformation the K-medoids method with the Partition Around Medoids (PAM) algorithm cluster the data streams.

Finally, paper [8] presents a strategy which is based on the independent processing of incoming data batches, through a preliminary summarization using histograms, followed by local clustering carried out on histograms, which ensures further summarization of the data. To track the proximity of data between data streams over time they used local clustering outputs to update the proximity matrix.

## 3. Time Series Data Streams Clustering Algorithms

### 3.1. Notations and Data Representation

A time series is an ordered sequence of values of a variable at equally spaced time intervals (e.g., 30 min electricity load readings). Let us assume that, $s_{j}={\left\{s_{j,1},s_{j,t},\ldots ,s_{j,n}\right\}}^{T}$ is a partial realizations from a j-th (j=1,…,m) real-valued processes $S_{j}=\left\{S_{j,t},t\in \mathbb{Z}\right\}$. Formally, the problem of grouping the time series data streams can be defined as follows. Let $S=\left\{s_{1}{}^{T},s_{j}{}^{T},\ldots ,s_{m}{}^{T}\right\}$ be the data stream composed of m time series each of length n (S is a matrix with m rows and n columns). For a l-th (l = 1,…,k) overlapping or non-overlapping time windows (blocks) with w time slots (intervals), $B_{l}$ (with partial realization $b_{j}={\left\{b_{j,1},b_{j,t},\ldots ,b_{j,w}\right\}}^{T}$) is a subset (of columns) of S, i.e., a matrix of dimension m×w (each block consists of a subset of times series from the same time interval. For a given block, $L_{l}\;=\;\left\{L_{l_{1}},L_{l_{o}},\ldots ,L_{l_{p}}\right\}$ represents a partition (of rows) of $B_{l}$ such that $L_{l_{o}}$ is the o-th cluster of $L_{l}$, $L_{l_{o}}\cap L_{l_{p}}\;=\;\emptyset ,\;\forall o\neq p$ and $\cup_{o=1}^{p}L_{l_{o}}\;=\;B_{l}$ [37].

**Figure 1:**
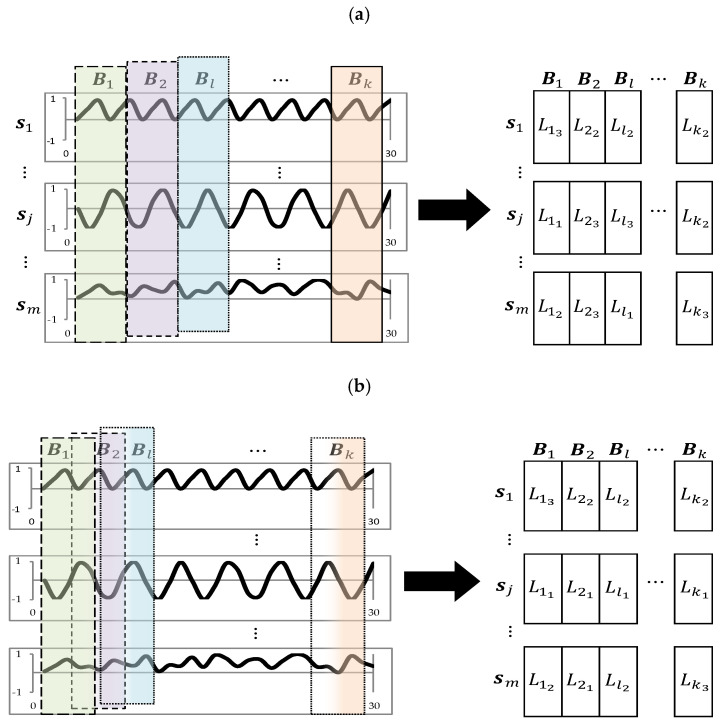
An exemplary data representation model with clustering: (**a**) Non-overlapping windows, (**b**) overlapping windows.

An exemplary data representations for overlapping (bottom part) and non-overlapping (upper part) windows with final clustering are depicted in Figure 1. On both figures on the left-hand side, there are m time series data streams, S, divided into k blocks each of length w (here w = 5). The right part of this figure illustrates an exemplary partition of the m time series from the l-th window ($B_{l}$) into $L_{l_{p}}$ cluster.

### 3.2. Histogram-Based Clustering Algorithm

The algorithm presented by [8] is composed of 4 main phases, where phases 1-3 are done online, while phase 4 is done offline. The goal of the phase 1 is to represent each time series data stream as a series of histograms by dividing the incoming data into (by default) non-overlapping time windows (this assumption will be further extended) and calculating the histogram of each l-th window:

(1) $$H_{j}^{l}=\left\{\left(I_{1},\pi_{1}\right),\ldots ,\left(I_{p},\pi_{p}\right),\ldots ,\left(I_{P},\pi_{P}\right)\right\},$$

where $I_{p}$ denotes P successive bins/intervals associated with the relative frequencies $\pi_{p}$ (weights), which sum up to 1. In this way, one can obtain, for each time window, a set of histograms which become the input for the local clustering procedure.

The purpose of the phase 2 is to get a local data partition (using BIRCH algorithm [29]) on a set of histograms that summarize the data behavior in each window. In order to do that the $L_{2}$ Wasserstein metric (distance) should be introduced, which simply calculate the distance between any two histograms $H_{k}^{l}$ and $H_{j}^{l}$. As shown in [46] this metric requires an initial homogenization step to ensure consistency of distance calculations, which is based on the histogram configurations. Since all histograms are uniformly dense in each $I_{p}$ interval, their quantile functions $Q_{j}^{l}$ are piecewise linear. Aforementioned homogenization step consists in dividing $Q_{j}^{l}$ functions in such a way that piecewise linear functions are defined on the same set of h cumulative probability values $q_{v}=\sum_{p=1}^{v}\pi_{p},\;\left(v=1,\ldots ,h\right)$ [8]. To make the computation faster, according to the authors [46], each bin $I_{v}=\left[\bar{I_{v}};\underline{I_{v}}\right]$ in the histogram can be represented as a function of a radius and a center, i.e., $I_{v}=\left[c_{v}-r_{v};c_{v}+r_{v}\right]$, where $c_{v}=\left(\bar{I_{v}}+\underline{I_{v}}\right)/2$ is the centre of each interval and $r_{v}=\left(\bar{I_{v}}-\underline{I_{v}}\right)/2$ is the radius. Finally, using this representation the $L_{2}$ Wasserstein distance is as follows:

(2) $$d_{W}^{2}\left(H_{k}^{l},H_{j}^{l}\right)=\overset{h}{\underset{v=1}{\sum }}\pi_{v}\left[{\left(c_{v}^{k}-c_{v}^{j}\right)}^{2}+\frac{1}{3}{\left(r_{v}^{k}-r_{v}^{j}\right)}^{2}\right].$$

The formula allows to take into account the features of two histograms being compared in terms of shape, range and location.

To perform a local clustering on l-th batch, aforementioned BIRCH algorithm requires two information about each o-th group (o=1,…,p), i.e., histogram centroid (average) $\bar{H_{o}^{l}}$ and $L_{2}$ Wasserstein-based variance $\sigma^{2}{}_{o}^{l}$. According to the [47] and based on the Formula (2), the mean of a set of histograms of equal frequency is obtained by the average of the centers and the average of the radii of the corresponding h intervals:

(3) $$\bar{H^{l}}=\left\{\left(\left[{\bar{c}}_{1}-\bar{r_{1}};{\bar{c}}_{1}+\bar{r_{1}}\right],\pi_{1}\right)\ldots \left(\left[{\bar{c}}_{v}-\bar{r_{v}};{\bar{c}}_{v}+\bar{r_{v}}\right],\pi_{v}\right)\ldots \left(\left[{\bar{c}}_{h}-{\bar{r}}_{h};{\bar{c}}_{h}+{\bar{r}}_{h}\right],\pi_{h}\right)\right\},$$

where:

(4) $${\bar{c}}_{v}=m^{-1}\overset{m}{\underset{j=1}{\sum }}c_{v}^{j};\;{\bar{r}}_{v}=m^{-1}\overset{m}{\underset{j=1}{\sum }}r_{v}^{j}.$$

On the other hand, a volatility measure for a set of histograms is the average of the $L_{2}$ Wasserstein measure between each j-histogram and the average histogram defined in Formula (3):

(5) $$\sigma^{2}{}^{l}=\frac{1}{m}\overset{m}{\underset{j=1}{\sum }}d_{W}^{2}\left(H_{j}^{l},\bar{H^{l}}\right).$$

The rationale in favor of this phase is to perform a single scan of the input data in order to obtain a division into a large number of clusters with low variability. To do that authors in [8] adopted the basic BIRCH algorithm to histogram-based data structures. Whenever a new time window is introduced, the algorithm allocates each $H_{j}^{l}$ histogram to existing micro-clusters or generates new micro-clusters according to a fixed threshold u that controls the growth of heterogeneity in micro-clusters. In other words, if the $L_{2}$ Wasserstein distance to the nearest micro-cluster centroid is smaller than the predefined threshold $d_{W}^{2}\left(H_{j}^{l},\bar{H_{o}^{l}}\right)<u$ then $H_{j}^{l}$ histogram (representation of the time series data stream) is assigned to this cluster, otherwise it creates entirely new cluster, with the initialized variance $\sigma^{2}{}_{o}^{l}$ set to at the $L_{2}$ Wasserstein distance to the nearest cluster.

In phase 3 an update of the proximity matrix $A^{l}=\left[a^{l}\left(k,j\right)\right]$ is performed, which registers the dissimilarities between the streams. The proximity matrix is updated incrementally (each cell $a^{l}\left(k,j\right)$) each time a new data window is processed in phase 2, therefore, it tracks the proximities over time, using information only from the local partitions. If two histograms $H_{k}^{l}$ and $H_{j}^{l}$ fall into the same micro-cluster the proximity matrix is updated by adding the value of the variance $\sigma^{2}{}_{o}^{l}$ of this cluster:

(6) $$a^{l}\left(k,j\right)=a^{l}\left(k,j\right)+\sigma^{2}{}_{o}^{l}.$$

On the other hand, if these two histograms fall into different micro-clusters, the cell is updated by adding the mean of two distances:

(7) $$a^{l}\left(k,j\right)=a^{l}\left(k,j\right)+\frac{d_{W}^{2}\left(H_{k}^{l},\bar{H_{o}^{l}}\right)+d_{W}^{2}\left(H_{j}^{l},\bar{H_{p}^{l}}\right)}{2},$$

i.e., $L_{2}$ Wasserstein distances to the nearest micro-cluster centroids for both histograms. This update strategy allows to use only information from the micro-clusters, thus it requires only $m^{2}/2$ operations.

Finally, phase 4 provides an ultimate global clustering of the time series data streams from $B_{l}$ block by grouping the updated proximity matrix into $L_{l}$. In order to obtain such partition DCLUST algorithm [48] is employed which minimizes intra-cluster variability, expressed by the sum of the dissimilarities between all pairs of elements within a cluster:

(8) $$\overset{p}{\underset{o=1}{\sum }}\underset{k,j\in L_{l_{o}}}{\sum }a^{l}\left(k,j\right)\;\to min.$$

According to the authors [8] histograms are fast to compute with the time complexity O(wP). The generation and the update of histogram micro-clusters, through a single scan of the histograms in a window, induces the time complexity of the algorithm is linear in m and p.

### 3.3. ClipStream Algorithm

The ClipStream algorithm is composed of two main phases [35], i.e., online data abstraction (representation) and an offline clustering. The first data representation phase includes a fast and incremental method of calculating feature vector from each $B_{l}$ block named FeaClip and automatic detection of outliers. The second offline phase aims at grouping of a new data abstraction, aggregation of time series data streams in the cluster and the change detection process.

The feature extraction approach from the first phase is based on a so called clipped representation. Let us first define a short window $b^{short}$ as a subsequence of an original time series data stream s of length z (z is shorter than window length w, and it could represent e.g., each day having 24 or 48 recordings; see also Section 3.1.) and a long window $b^{long}$ which consists of last d consecutive short windows (therefore it is of length d∗z). Next, a new representation (with reduced dimensionality p<z) of $b^{short}$ is $repr^{short}$ defined as below, first:

(9) $${\hat{b}}_{t}^{short}=f\left(x\right)=\{\begin{array}{c} 1\;\;\mathrm{if}\;\;\;b_{t}^{short}>\mu \\ 0\;\;\;\;\;\;\;\;\;\mathrm{otherwise} \end{array},$$

${\hat{b}}^{short}$ is a clipped (bit-level) abstraction of the original block, where μ denotes a mean value of $b^{short}$. Then, the compression method called Run Length Encoding (RLE) [49] is applied on this abstraction to create the final representation $repr^{short}$ (of length 8) defined as:

(10) $$repr^{short}=\left\{\begin{array}{c} max_{1}=max.\;\mathrm{from}\;\mathrm{run}\;\mathrm{lengths}\;\mathrm{of}\;\mathrm{ones},\\ sum_{1}=sum\;\mathrm{of}\;\mathrm{run}\;\mathrm{lengths}\;\mathrm{of}\;\mathrm{ones},\\ max_{0}=max.\;\mathrm{from}\;\mathrm{run}\;\mathrm{lengths}\;\mathrm{of}\;\mathrm{zeros},\;\;\\ crossings=\mathrm{length}\;\mathrm{of}\;\mathrm{RLE}\;\mathrm{encoding}\;-1,\\ f_{0}=\mathrm{number}\;\mathrm{of}\;\mathrm{first}\;\mathrm{zeros},\\ l_{0}=\mathrm{number}\;\mathrm{of}\;\mathrm{last}\;\mathrm{zeros},\\ f_{1}=\mathrm{number}\;\mathrm{of}\;\mathrm{first}\;\mathrm{ones},\\ l_{0}=\mathrm{number}\;\mathrm{of}\;\mathrm{last}\;\mathrm{ones}, \end{array}\right\}$$

Finally, the ultimate $repr^{long}$ abstraction is an union of d short representations $repr_{d}^{short}$ which has length d∗8. Whenever a new window $b_{d+1}^{short}$ is arrived, first 8 features from $repr^{long}$ are removed and new $repr_{d+1}^{short}$ is attached to the end of $repr^{long}$.

Based on the calculated FeaClip abstractions of all available time series data streams, outlying values can be easily and automatically detected by using domain knowledge. To automatize this, mean values of crossings and $sum_{1}$ are calculated for each stream and corresponding $repr^{long}$. Bead on these statistics, lower and upper quartiles and IQR (interquartile range) are calculated to create box-and-whisker diagrams, with threshold value λ set at 1.5. Time series with the characteristics that meet the following conditions: $Q_{1}^{sum_{1}}-\lambda *IQR^{sum_{1}}\leq x\geq Q_{3}^{sum_{1}}+\lambda *IQR^{sum_{1}}$ and $x\geq Q_{3}^{crossings}+\lambda *IQR^{crossings}$, are considered as non-outliers. Outlying values are not deleted from the whole clustering, they are simply stored in memory, and after the clusters are determined, those objects are assigned to the nearest ones.

Once the data representation phase is completed second offline stage follows to create the final grouping. Only filtered (without outliers) $repr^{long}$ representations are subject to clustering using K-medoids method with Partition Around Medoids (PAM) algorithm [50] with Euclidean distance. To capture the dynamic and evolving nature of time series data streams, the number of clusters should also be determined dynamically. Therefore, the optimal number of clusters is determined on the basis of the internal measure of Davies–Bouldin index [51]. During the first iteration of clustering the number of possible clusters is determined in the range $p_{min}-p_{max}$, where p that minimizes the Davies–Bouldin index is chosen. To speed up further iterations of clustering the optimal number of clusters is selected from 〈p−2,p+2〉, where p is the number of clusters from the previous iteration.

In order to carry out the process of grouping time series data streams only when it is necessary, i.e., only when data streams evolve and change of distributions occur, a stage for detecting concept drift is conducted. It detects changes of the Empirical Distribution Function (EDF) of the normalized aggregated data stream within each cluster, using K-sample Anderson–Darling test, defined as:

(11) $$A_{kw}^{2}=\frac{1}{w}\overset{K}{\underset{k=1}{\sum }}\frac{1}{z}\overset{w-1}{\underset{t=1}{\sum }}\frac{{\left(wN_{kt}-tz\right)}^{2}}{t\left(w-t\right)},$$

where (according to the Section 3.1 and notation introduced at the beginning of this section) $s_{j,t}$ is the t-th recording in the k-th sample, $N_{kt}$ denotes the number of observations in the k-th sample that are not greater than $x_{t}$, where $x_{t}<\cdots <x_{w}$ is the pooled ordered sample (long window). Concept drift is detected if p-value is less than the significance level α set at 0.05, however clustering is updated only if one of these conditions are meet: (1) The number of detected changes is more than half of the grouped p time series (number of clusters); (2) the number of detected changes is higher than in the previous step of the sliding window.

According to the authors [35] the representation phase has the linear time complexity O(w) with respect to the length of the time window. Outlier detection phase is linear O(m). The offline phase consists of the PAM clustering algorithm that for each iteration has the quadratic complexity of $O\left(p{\left(\left(m-m_{o}\right)-p\right)}^{2}\right)$, where $m_{o}$ denotes number of outliers.

### 3.4. Extended TS-Stream Algorithm

The algorithm presented by [45] is an extended (improved) version of the algorithm presented in [37]. In general, it evokes a model with a structure similar to the decision tree, but built in an unsupervised manner. The top-down strategy is employed to build the tree, starting from all times series data streams in the same main cluster (root) and gradually creating partition or aggregations. Each indirect node executes a binary test of a type $feature_{value\;}\leq x$ for a specific time series descriptive measure. Once a leaf is reached, the time series is stored together with other time series which belong to the same leaf.

During the first step the algorithm calculates descriptive measures (here also called coefficients, characteristics, indices) for each time series data stream. This gives a matrix of characteristics of the dimension m×f, where f is the number of characteristics. To make all features comparable (which is required when variance minimization criterion is used), for each column of the matrix the z-score normalization of the form x=(x−μ)⁄σ is performed. A simple and natural way to model each time series data stream is to use generating functions to depict their behavior in time domain. Unfortunately, many of the existing grouping techniques do not take into account specific characteristics of the generating function, e.g., stochasticity, linearity, and stationarity. So, the algorithm employs many descriptive measures in order to obtain the appropriate characteristics of the generating function to better describe the resemblance between the series.

Authors in [37] claim that after their investigation of several descriptive measures such as Discrete and Continuous Wavelet Transforms, Recurrence Quantification Analysis measures, Empirical Mode Decomposition, Lyapuno, Discrete Cosine Transform, Detrended Fluctuation, Autocorrelation function and Box and Jenkins model parameters, the best ones were Hurst exponent, Auto Mutual Information (AMI) and Discrete Fourier Transform (DFT). Those indices have been chosen because they are efficient to compute and provide high information gain (see Formulas (12)–(14), below).

The Hurst’s exponent, is a measure of long-term memory of the time series. It refers to the auto-correlation of the time series and the rate at which it decreases as the delay between value pairs increases. There are different estimating approaches of the exponent; the Scaled Range approach is most often used. The Hurst, H exponent is defined in terms of the asymptotic behavior of the Scaled Range as a function of the time series time interval, as follows [37]:

(12) $$\frac{R_{t}}{S_{t}}=ct^{H},$$

where t stands for the time span of the observation, c is a constant, $R_{t}$ is the range of the first t cumulative deviations from the mean, and $S_{t}$ is their standard deviation.

The second measure, which is Auto Mutual Information (AMI), provides insight of how much one random variable explains the other variable. To calculate this characteristic, a histogram (with intervals) has to be created. Let $p_{i}$ be the probability that the signal has a value inside the i-th intervals, and let $p_{ij}\left(\tau \right)$ be the probability that $s_{t}$ is in intervals i and $s_{t+\tau }$ is in intervals j. Then, the AMI for time delay, τ, is defined as [37]:

(13) $$AMI\left(\tau \right)=\sum p_{ij}\left(\tau \right)\log\left(\frac{p_{ij}\left(\tau \right)}{p_{i}p_{j}}\right).$$

The last one is the Discrete Fourier Transform (DFT) [52] which describes time series in the frequency domain. This transform, after receiving a time series $s_{t}$ as input, provides a new series $X_{m}$ of n complex numbers, each one describing a sine function at a given frequency [37]:

(14) $$DFT=X_{m}=\overset{n-1}{\underset{t=0}{\sum }}s_{t}e^{-j2\pi tm/n},$$

where $j=\sqrt{-1}$. The Fourier transform helps to characterize the generating function of this time series by indicating the most relevant frequencies, i.e., first 20 DFT coefficients of every time series in each window with the highest energy have been retained.

To split the times series into different clusters/nodes, each time a dedicated function is called which is accountable for finding the best coefficient for the binary test of the current node. This function takes as its input normalized matrix of characteristics and aims to minimize the weighted variance criterion of the form:

(15) $$Gain=\sigma^{2}\left(V\right)-\frac{n_{left}*\sigma^{2}\left(V_{left}\right)+n_{right}*\sigma^{2}\left(V_{right}\right)}{n},$$

where V is the current node consisting of n time series data streams, $\sigma^{2}\left(\cdot \right)$ is the variance function, $V_{right}$ and $V_{left}$ are the nodes established after the split, each with $n_{right}$ and $n_{left}$ series, respectively.

In each consecutive iteration after obtaining a new time window the algorithm maintains the current tree model (structure from the previous iteration) and clusters time series based on the new batch of data. After this, the update stage begins, in which the breakdowns and/or aggregations are checked and executed, if necessary and/or possible [37], which is controlled by a set of parameters, i.e., α∈[0,1], λ∈[0,1], and minSeries. Two sibling leaves (denotes as LeftChild and RightChild) must be aggregated if their weighted variance (denoted as WVC) is greater than or equal to λ of the parent node variance (VP) computed from its test feature. This makes the structure of the tree simpler and more resistant to noise/outliers. If aggregation did not occur the algorithm checks for possible leaf splits, which is done if the weighted variance of its potential children decreases by at least α times its variance. Finally, to prevent a split when two possible children have less than a certain percentage of all observations, minSeries parameter controlling the complexity/depth of the tree is set by default at 5%.

The overall time complexity is $O\left(m^{2}w\right)$. It is important to note that the quadratic term in the algorithm refers to the number of time series, which is typically low (order of tenths) [45].

## 4. Research Framework and Settings

### 4.1. Numerical Implementation

As presented below, numerical experiments were prepared using *R* programming language working on Ubuntu 18.04 operating system on a personal computer equipped with Intel Core i7-9750H 2.6 GHz processor (12 threads) and 32 GB of RAM.

The first algorithm, which is histogram-based clustering, was implemented using several libraries. To represent each time series as a histogram and to compute the $L_{2}$ Wasserstein distance the *HistDAWass* package was used [47], which implements a framework of Symbolic Data Analysis, a relatively new approach for the statistical analysis of multi-valued data. Next, to get a local data partition based on a set of histograms a modification of *BR_BIRCH* package was used [53]. Finally, a *symbolicDA* [54] package was utilized to obtain a global clustering using DCLUST algorithm. The second algorithm, which is ClipStream, was entirely implemented using *ClipStream* library which is a software strictly connected to the article [55]. Finally, the extended TS-Stream algorithm was implemented in line with the following work [45].

### 4.2. Algorithms Parameters Setting

In order to have robust and consistent results all algorithms parameters settings are in line with the source articles and libraries. Since for the extended TS-Stream algorithm the parameters α and λ have a similar influence, it is not recommended to set one value as a function of the other. During the research preparation stage, it was observed that setting these two parameters to values smaller than 0.6 resulted in almost no splits. On the other hand, values greater than 0.6 could result in a too wide and too deep tree. Next, minSeries parameter which is responsible for controlling the size of a tree, is set at 5% (50 time series). Due to the fact that there are 1000 time series in the investigated data set (see Section 5) the final tree structure might have up to 20 leaves, i.e., clusters. 

For ClipStream algorithm, long ($b^{long}$) and short windows ($b^{short}$) length were set to 1008 or 48 for overlapping windows and to 1440 or 48 for non-overlapping windows (see Section 4.4), while threshold value λ determining outliers was set at 1.5. The optimal number of cluster derived by the Davies–Bouldin measure was determined in the range 5 and 11. The latter number was determined as an average number of clusters obtained for each batch (for both overlapping and non-overlapping windows) for extended TS-Stream algorithm. Finally, concept drift is detected if p-value is less than the significance level α set at 0.05.

Histogram-based clustering algorithm has the following changeable parameters: P, which determines number of bins for each histograms, was set at 10 (average number of clusters obtained for both aforementioned algorithms), u, which is a threshold on the micro-cluster size, was set at 0.01, and because other two remaining algorithms usually provided maximal number of clusters, o parameter, which defines number of clusters, was set at 11.

### 4.3. Tested Changeable Components

One of the main goals of the article is to find the best clustering algorithm and, if possible, to propose some improvements with regards to different components adopted from other algorithms. To do so, firstly, a comparative study between overlapping windows and non-overlapping windows was be conducted, i.e., research was conducted in two different variants (see also Figure 1):

- Using non-overlapping window: This approach is in line with our previous study where the window length w of each block $B_{l}$, has been set to 30 days. As the electricity consumption data were recorded at 30-min intervals, each window has length of 1,440 (2 × 24 h × 30 days);
- Using overlapping window: This approach is in line with the article [35] implementing ClipStream algorithm where window is of length 21 days (3 weeks). In this case, each time there are two overlapping weeks led by the new arriving week (2 × 24 h × 21 days = 1008).

Secondly, a new Fast Fourier Transformation based features (calculated in liner time) is proposed, allowing to compress and represent time series using the business context. In our previous paper a set of 20 dominating Fourier coefficients have been taken as descriptive measures (see also Section 3.4). To make the usage of Fourier coefficients more intuitive, in this paper, the frequency domain have been divided into four intervals/ranges. Each of them represents electricity consumption behavior changes, respectively, monthly, weekly, daily, and all more frequent (see Table 1). The frequency is calculated with respect to the following equation:

(16) $$f_{c}\left(m\right)=\left(f_{s}*m\right)/w,$$

where $f_{c}\left(m\right)$ is the frequency of m-th coefficient $f_{s}$ is the frequency of sampling, w number of samples (i.e., window length) used in Fourier transform. A period is calculated as $1/f_{c}\left(m\right)$. As it can be noted, end of an interval is not a beginning of the another one. One should remember about discrete nature of values of DFT coefficients. Moreover $f_{c}\left(0\right)$ represents the mean value.

Those aforementioned features were used in the extended TS-Stream algorithm (in this case a node partition is performed based on only one feature) and in the ClipStream algorithm. In the latter case instead of FeaClip representation each time series is represented base on those 4 features.

Thirdly, to conduct process of time series data streams clustering only when it is necessary, a stage for detecting concept drift using K-sample Anderson–Darling test (idea taken from ClipStream algorithm) was also implemented in the extended TS-Stream algorithm. 

Finally, it is necessary to mention that all above improvements/components were not implemented in the histogram-based clustering algorithm, because it would entirely change the logic and the behavior of this algorithm.

### 4.4. Framework and Measures for Clustering Comparison

The main problem existing in the investigated area is the fact that there are no explicit frameworks, measures, criteria allowing to assess the performance, effectiveness and to compare algorithms to each other. To overcome this issue, we have proposed the following framework.

To compare the results of the grouping against external criteria, a measure of consensus is needed. Since it is assumed that each time series is assigned to only one cluster a natural way is to utilize the Adjusted Rand Index which is a measure of the similarity between two data clusterings. However, the practical aim in this article is to propose an optimal tariff for each time series. In this context we would like to know which clustering algorithm provides stable results, i.e., clusterings that are similar to each other. To do so we reformulated standard ARI measure as follows:

(17) $$ARI=\frac{\sum_{uo}\left(\begin{array}{c} n_{ou}\\ 2 \end{array}\right)-\left[\sum_{o}\left(\begin{array}{c} n_{o*}\\ 2 \end{array}\right)\sum_{u}\left(\begin{array}{c} n_{*u}\\ 2 \end{array}\right)\right]/\left(\begin{array}{c} n\\ 2 \end{array}\right)}{\frac{1}{2}\left[\sum_{o}\left(\begin{array}{c} n_{o*}\\ 2 \end{array}\right)+\sum_{u}\left(\begin{array}{c} n_{*u}\\ 2 \end{array}\right)\right]-\left[\sum_{o}\left(\begin{array}{c} n_{o*}\\ 2 \end{array}\right)\sum_{u}\left(\begin{array}{c} n_{*u}\\ 2 \end{array}\right)\right]/\left(\begin{array}{c} n\\ 2 \end{array}\right)},$$

where $n_{ou}$ denotes the number of objects that are in both, cluster $l_{o}$ form l-th time window and cluster $l_{u}$ from the l+1 time window ($l_{u}$ is simply the same cluster as $l_{o}$ but from consecutive window), with the marginal distributions denoted as $n_{o*}$ and $n_{*u}$. After comparing each batch to each other an upper triangle matrix is created [45] (for an example please see Table 4). 

The second measure is closely related to the selection of an optimal tariff for each customer. Let us assume that a particular customer has a base tariff G11 (single time zone with flat price rate per kWh) over an entire year. From the customer perspective it might be better to change a tariff to G12 for an entire year. Furthermore, one may analyze more frequent changes of the tariff e.g., after each month or even after each week. To answer that question we propose the following approach:

(1) For a particular time window l apply a given clustering algorithm;
(2) Assign a particular customer to his cluster;
(3) Determine an optimal tariff for the entire cluster, i.e., the lowest price for an aggregate consumption of all customers in cluster by calculating the total electricity cost if they would belong to G11, G12, G12r or G12w tariff plan;
(4) Select an optimal tariff from the previous step as an optimal tariff for a given customer;
(5) Deploy an optimal tariff for each customer as a tariff for the next time window l+1;
(6) Return to the first step.

According to the above procedure it might happen that for a given customer an optimal tariff for an entire year is G12. However, on the other hand it might happen that an optimal tariff will change after each time a new batch of data arrives. Next, to assess whether application of a particular clustering algorithm and aforementioned procedure make sense, we propose to derive, as previously, a similar upper tringle matrix having the following values:

(18) $$\mathrm{Tariff}\;\mathrm{improvement}=\frac{\mathrm{dynamic}\;\mathrm{optimal}\;\mathrm{tariff}}{\mathrm{static}\;\mathrm{optimal}\;\mathrm{tariff}}.$$

To clarify that, let us consider first data batch l in a given year (for non-overlapping windows there would be 12 batches). This case is represented as the first top row in the upper triangle table (Table 4). Based on that particular window it was decided that an optimal tariff for the entire year is G12w (an optimal tariff for a cluster where a particular customer belongs), therefore, for this investigated row, denominator in the above equation takes always the same value, i.e., price of this fixed tariff for a particular customer calculated for each month separately. On the other hand, nominator is determined dynamically. For the first column it takes the same value as the denominator. For the remaining eleven columns (batches from l+1,…, l+11) it takes dynamically changeable price of the tariff determined in the 5th step of the mentioned earlier procedure. Such table is prepared for each customer, therefore to have only one global table, as in case of the ARI, each field in the final table was calculated as the mean value of the 1000 customer-wise matrices.

The last measure is the weighted volatility of time series for a given block $B_{l}$. After the division, the time series are spread over several groups. It is assumed that the variation (standard deviation) of electricity consumption in each group is to be less than the variation of time series in only one group (root) [45]. Furthermore, because of the difference in the size of each group, the measure takes into account this fact by assigning smaller weights to a smaller leaf—as in the right-hand side of the Equation (19):

(19) $${\mathrm{Weighted}\;\mathrm{volatility}}_{B_{l}}=\underset{L_{l_{o}}\in L_{l}}{\sum }\frac{\#L_{l_{o}}}{m}*\sigma \left(L_{l_{o}}\right),$$

where $\#L_{l_{o}}$ denotes the number of time series for a given cluster, m denotes the number of time series in a block $B_{l}$ and σ(·) is the standard deviation of all times series assigned to a given cluster $L_{l_{o}}$.

## 5. Empirical Analysis

### 5.1. Data and Tariffs Characteristics

The dataset used in this research is originated from the Irish Commission for Energy Regulation (CER) project where the measurements of the electricity load where recorded for 4182 households between July 2009 and December 2010. In total, time span covers 75 weeks where each reading was recorded with 30 min data granularity [5]. Due to the missing recordings in the time series and computational complexity of the investigated algorithms, the research was conducted using data from 1000 households selected randomly.

Unfortunately, CER dataset does not provide any information regarding tariff plan of each customer. After investigation of several tariffs plans provided by electricity suppliers in the European countries, it can be stated that there are many similarities. Therefore, to conduct simulation of the optimal tariff, all the information and the tariff prices were taken from one of the biggest energy holding company in Poland.

**Figure 2:**
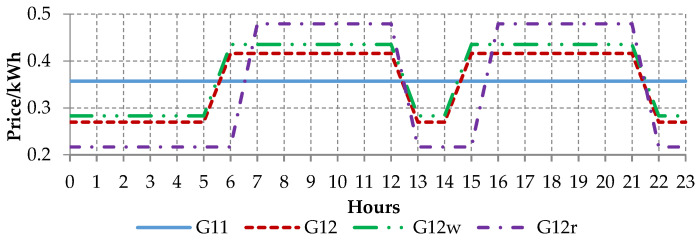
Prices in G11, G12, G12r, and G12w tariff plans (1 Polish PLN~0.22 EUR).

Depending on the tariff plan, the customers can benefit from lower prices per kWh if the usage falls between certain time zones. In Figure 2 the prices for G11, G12, G12w, and G12r tariff are presented. G11 tariff (blue straight line) has the fixed price of 0.35 PLN/kWh. G12r tariff (purple dotted-dashed line) plan has lower rate of 0.21 PLN/kWh between 10 p.m. and 7 a.m. and between 1 p.m. and 4 p.m., while the higher rate of 0.48 PLN/kWh is applicable outside these windows. G12w tariff (green double dotted–dashed line) has lower rate of 0.28 PLN/kWh during the weekends and Monday–Friday between 10 p.m. and 6 a.m. and between 1 p.m. and 3 p.m., while the higher price of 0.43 PLN/kWh is applicable outside these windows.

Let us now simulate what is the relation between the best and the worst tariff for each customer. Table 2 shows various statistics of the simulation (aggregated over 1000 customers) for non-overlapping windows case. When dynamically changing an optimal tariff for each customer a minimal improvement between the best and the worst individual tariff is 2.39%, while the biggest improvement reaches 19.27%. Second row of the table shows what is the improvement between dynamically changing an optimal tariff and one fixed optimal tariff derived based on the entire period. It was observed that dynamic change resulted in average improvement of 0.28%. Finally, it can be concluded that, on average, an optimal tariff would change almost 5 times, out of 17 data batches, each 30 days long, in the analyzed timeframe.

When speaking of overlapping windows case (Table 3), results are slightly higher. Average improvement between the best and the worst individual tariff for each batch increases to 8.47%, while the best individual tariff for each batch vs best individual tariff for the entire period increases to 0.51%, on average. Due to the fact that there are 73 batches in this scenario, each batch of 21 days long, the median of dynamic individual tariff changes is 25.

Those results present the best and the worst case scenarios, when an optimal tariff is derived for each customer separately without any clustering algorithm. Therefore, those results provide benchmarking ranges between which the clustering results presented in the following subsections will be included.

### 5.2. Clustering Results

Let us now investigate which algorithm provide relatively robust results, i.e., overall groupings that are similar to each other (in other words, maintaining time series belonging to the same clusters). For the non-overlapping case, the extended TS-Stream algorithm provides on average 11 clusters, all having more than 5% of all time series. For the 17 investigated batches on average each time series should change his optimal tariff 7.98 times (median is 8; this is determined as the optimal tariff for the cluster to be monitored). The ClipStream algorithm changes the tariff 5.38 times on average (median is 6), while not using the concept drift results in increasing these values to 6.52 and 7. On average, histogram-based algorithm changes the tariff 7.04 times (median is 7). All aforementioned numbers are higher than those reported in Table 2, where the best tariff is chosen separately for each customer without any clustering algorithm, which means that a time series changes its tariff more frequent than it should. For better understanding of the idea, in this article we present only sample matrix of the ARI index obtained for the ClipStream algorithm (in Table 4). Table 5 and Table A1 (in the Appendix A) provide various statistics of the ARI and tariffs improvement derived based on the upper-triangular matrixes (described also in Section 4.4) for both non-overlapping and overlapping windows (see Appendix A). 

In this example, similarity (measured using ARI) between the first batch $B_{1}$ and second the batch $B_{2}$ is 0.100. Clustering from the first batch is the least similar to batches from seven to nine (0.040). Because algorithm detected no concept drift between batches $B_{7}$–$B_{9}$, the change of clusters membership did not occur which results in ARI equals 1.

According to the results presented in Table 5 (the best results for each statistic are bolded), it can be seen that, on average, the highest ARI provides histogram-based algorithm. This is impacted by two things, first—it always generates the same number of clusters. Secondly, it divides customers into the clusters based on the iteratively updated (after each batch) global proximity matrix which uses partition from the BIIRCH algorithm (second step of this algorithm). This step provides only a minor modification of the global matrix and once in the last step the DCLUST is incorporated, it provides very similar groupings (customers rarely change their cluster). It should be noted that whenever ClipStream algorithm decides not to make any changes ARI is equal to 1. The worst results are connected with the extended TS-Stream algorithm (median is 0.033).

For the overlapping widows case (Table A1 in the Appendix A) the dependencies are similar. One more time the histogram-based algorithm produces the most stable partitions. In previous case, for the extended TS-Stream algorithm concept drift module was not used. This time for couple of batches the tree preserved the same structure which increased the highest value at 0.326. What is interesting, for ClipStream algorithm the new data representation (Fourier coefficients) increases lower (up to median) statistics.

In the similar manner as for the ARI index the upper triangle matrix has been derived for the tariffs improvement (Equation (18)).

From practical point of view it is better for the electricity provider to have customer groups with relatively similar size [2]. The extended TS-Stream algorithm guaranties that each cluster has no less than 5% of all customers, and after investigation of the group size it can be stated that this algorithm produces clusters with the similar size. On the other hand, both ClipStream and Histogram-based algorithms do not have such restriction. On average, ClipStream algorithm generates one (rarely two) cluster having only couple of customers (1–5 time series). Histogram-based algorithm usually produces three up to four clusters whose are very small. This observation has high influence on the values of the investigated metrics (they are rewarded), since in small groups memberships change rarely and the volatility is small (see Table 6 and Table A3).

According to the results presented in Table 6, the least volatile partitions provides the extended TS-Stream algorithm, median is 21.06 while mean is 23.22 (since there were no batches when the concept drift module was used both versions produce the same results). Seconds place in this ranking takes the Histogram-based algorithm whose maximal volatility is even smaller than for the extended TS-Stream. For the overlapping windows case, the least volatile groups produces the histogram-based algorithm. Slightly worse results are connected with the Extended TS-Stream (with the concept drift module) whose the minimal statistic is even smaller than for the histogram-based algorithm. Finally, in both windows (overlapping and non-overlapping), new data representation and not use the concept drift procedure in ClipStream worsen the results.

### 5.3. Tariff Evaluation

In this section tariff improvements are discussed. When it comes to the various statistics for non-overlapping windows, it is observed that the all investigated algorithms provide, on average, an improvement of 0.3%–0.4%, please refer to Table 7. The highest improvement is observed for the Extended TS-Stream and the histogram-based algorithms, and for the ClipStream algorithm with the newly proposed data representation (up to 1.8%). Moreover, the first two algorithms mentioned do not produce worse results (please refer to the first column with Min values).

For the overlapping windows case, please refer to Table A2, one more time, all algorithms usually provide the improvement, with the mean value between 0.1% and 0.2%. Unfortunately, in the worst-case-scenario each algorithm chose worse tariff, the smallest worsening (−0.1%) is for the extended TS-Stream algorithm without concept drift module.

The last results presented below are to answer the question, whether it is possible and justified to use clustering (and associated optimal tariffs for each group) obtained for a particular batch $B_{l}$ and the deploy those optimal tariffs as the applicable tariffs in the following period $B_{l+1}$. Table 8 and Table A4, provide statistics of the tariffs improvement compared to the basic (flat) tariff G11 in case when the future optimal tariff for each customer (for the next data batch) is derived as the current optimal tariff for the cluster to which a particular customer belongs. The advantage of this approach is that it does not require training nor the use of any predictive models.

As shown in Table 8, for the non-overlapping windows case, on average, it is possible to achieve some improvement. The ClipStream algorithm provides better results of 0.31% comparing to the base tariff (removing concept drift module gives improvements as well). The mean improvement for both versions of the extended TS-Stream produces no improvement; however, median value equals −0.09%. Unfortunately, the histogram-based algorithm usually provides worse tariff than costs related with the G11. It should be noted that when comparing the optimal predicted tariff to the random tariff (rather than to the G11), on average, the results are always better (see Table A5). For the extended TS-Stream algorithm it is 1.66%, for the ClipStream algorithm (base version) it is 2.17%, and for the histogram-based algorithm it is 1.50%.

For the overlapping windows case (batch size equals 3 weeks while each time new data cover one week), please refer to Table A4, the improvements are more common and clear for all algorithms, i.e., according to the median and to the mean value the improvement is positive. Only for the statistics such as 3rd quartile and above the worsening can be noted. The biggest improvement is noted for the base version of the ClipStream algorithm (7.9%). Second place in terms of the mean value belongs to both versions of the extended TS-Stream algorithm (0.21%; 0.20%).

Finally, when it comes to the comparison to the random assignment of tariff (as an optimal for the future), the extended TS-Stream algorithm (base version) achieves improvement of 2.69%, for the ClipStream algorithm (base version) it is 2.91%, and for the histogram-based algorithm it equals 2.65% (see Table A6).

Based on the results we could summarize the comparative study between overlapping windows and non-overlapping windows and their impact on the choice of an optimal tariff as outlined in Table 9. For the purpose of results discussion the average improvements were considered. It was observed that the implementation of the current best tariff is feasible and could deliver the benefits for both, overlapping and non-overlapping windows. Specifically, for non-overlapping windows the general tariff improvement was up to 0.40%, on average, depending on the algorithm. In case of tariffs improvement comparing to the G11 tariff plan the highest improvement was for overlapping windows, where two ClipStream algorithms (with and without concept drift) were able to deliver up to 0.43% of the improvement, on average.

Importantly, the results, in terms of the tariff improvement, are only the highlight for possible knowledge utilization based on the algorithms that were used for profiling the customers. Nevertheless, the results are promising although the improvements might appear negligible. Please note that the improvement rates of 0.40–0.43%, as provided in Table 9, directly influence the elasticity of electricity demand. In case of Poland, the whole installed capacity of the system is approx. 45,000 MW so the improvement of 0.43% is representing 193.5 MW which is an equivalent of one power block in the power plant. Therefore, if some of the usage can be shifted outside peak hours then the benefit is not only for the customers but also for the electricity operators who can purchase the electricity cheaper. 

### 5.4. Other Applications—Australian Case Study

To proof the applicability of the dynamic profiling approach further analysis was conducted based on the data from the customer trial conducted as part of the Smart Grid Smart City (SGSC) project [56]. It provides sets of customer time of use (half hour increments) and demographic data for Australia between 2010 and 2014. For the purpose of the case study 998 households were randomly extracted covering 1 September 2012–28 February 2014 time frame. The reason to select that time frame was availability of complete data, i.e., without missing values. In total, 25,399 data points were analyzed, each representing half hour readings. 

For the purpose of results discussion the average improvements were considered as presented in Table 10. It was observed that the implementation of the current best tariff is feasible and could deliver the benefits for both, overlapping and non-overlapping windows. Specifically, for non-overlapping windows the general tariff improvement was up to 0.96%, on average, depending on the algorithm. In case of tariffs improvement comparing to the G11 tariff plan the highest improvement was for overlapping windows, where two ClipStream algorithms with and without concept drift, were able to deliver up to 1.08% and 1.06% of the improvement, on average, respectively. The results are consistent with the results on Irish data set. However, this time an improvement is considerably higher what can influence directly the elasticity of electricity demand. 

More detailed analysis are presented in Appendix B, please refer to Table A7, Table A8, Table A9, Table A10, Table A11, Table A12, Table A13, Table A14, Table A15 and Table A16.

### 5.5. Other Applications—London Case Study

Another verification of dynamic profiling approach was conducted based on the data from UK Power Networks led Low Carbon London project [57]. The dataset contains energy consumption in kWh (per half hour) for the sample of 5567 London households observed between November 2011 and February 2014. The customers in the trial were recruited as a balanced sample representative of the Greater London population. 

For the purpose of the case study 1000 households were randomly extracted covering 1 September 2012–28 February 2014 time frame. The reason to select that time frame was availability of complete data, i.e., without missing values. In total, 25,440 data points were analyzed, each representing half hour readings. 

To enable comparison of the results with previous applications (case studies) the average improvements were considered, as presented in Table 11. It was observed that the implementation of the current best tariff is feasible and could deliver the benefits for both, overlapping and non-overlapping windows. Specifically, for non-overlapping windows the general tariff improvement was equal, on average, to 0.93% for Extended TS-Stream without concept drift. The lower improvements, between 0.15% and 0.39%, were observed for other algorithms. In case of tariffs improvement comparing to the G11 tariff plan the highest improvement was for overlapping windows, where histogram-based approach resulted in the improvement of 0.68%, on average. Other methods were able to deliver improvements between 0.49% and 0.65% which could be considered significant, too. The improvement for non-overlapping windows was slightly lower, i.e., 0.55% and similarly, it was observed for histogram-based clustering approach. The results are consistent with the results on Irish data and Australian data. 

More detailed results are presented in Appendix C, please refer to Table A17, Table A18, Table A19, Table A20, Table A21, Table A22, Table A23, Table A24, Table A25 and Table A26.

## 6. Conclusions

Data streams clustering is one of the most common ways of analyzing data that is potentially infinite and evolves over time. Although the literature provides some methods of the data streams clustering, unfortunately, majority of them are not appropriate for the whole time series data streams clustering. Even though electricity consumer objectives are usually based on monetary benefits, electricity providers benefit from the knowledge of consumer’ profiles, to create individualized means aimed at consumers with compatible use profiles and socio-economic behavior. The analysis has shown that there are prominent distinction between consumers’ behaviors, which allows us to distinguish homogeneous groups. 

Through the CER Irish data analysis and two other case studies, i.e., Australian and London data sets, an attempt was made to evaluate different ways of time series data streams clustering by comparative study of the state-of-the-art algorithms, as well as new combinations employing elements from different algorithms. From the technical point of view the results introduce a general guidance on when and where to apply a particular clustering algorithm (along with its improvements).

It was revealed that the extension to the way of ARI index calculation (and its statistics) based on the upper triangle matrix, which compares blocks to each other, provides good evaluation framework, and it also allows to visualize the dependencies. This part of the research has shown that the best results, in terms of the similarity of the clusters, are provided by the histogram-based clustering algorithm. That is due to the fact that the algorithm always performs a partitioning using the same number of clusters and the underlying procedure is less fragile to any distribution changes than other two algorithms. Therefore, if the electricity providers need stable partitions this algorithm would be their first choice.

Furthermore, to obtain a partition which provides clusters with the least weighted volatility the extended TS-Stream algorithm should be applied. It is mainly caused by the fact that this algorithm is able to expand or to shrink the tree structure very quickly according to the distribution changes of the particular phenomenon. On the other side of the pole is the ClipStream algorithm.

As it was presented in our previous work [45], standard TS-Stream algorithm outperforms benchmarking clustering methods and, in addition, this research indicates that these results can be further improved. The new Fast Fourier Transformation based features allow to improve the operation of the base for this algorithm. The new data representation slightly deteriorates the performance of the ClipStream algorithm; however it should be noted that this time a business interpretation is prevailing. Moreover, a much smaller dimension is needed to represent a given time series, i.e., only 5 features instead of 8 multiplied number of weeks (3 weeks for overlapping and 4 weeks for non-overlapping windows).

In terms of the implementation/software requirements all the algorithms are able to work in linear time, however the histogram-based algorithm requires $O\left(m^{2}\right)$ memory space. It also produces fixed number of clusters. For the ClipStream algorithm it is necessary to set up minimal and maximal number of cluster in advance (which sometimes might be impracticable or unfounded). The extended TS-Stream algorithm is the most flexible in its nature what allows to incorporate new descriptive measures, data representation and concept drift detection module.

When it comes to the comparison between overlapping and non-overlapping windows, as it might expect, statistics of the ARI and weighted volatility for the overlapping windows are usually better (base version of each algorithm). This is due to the fact that each time we analyze almost the same time series that differ only with one new added week.

Based on the comparative study between the state-of-the-art time series data streams clustering algorithms and their modifications we could perform the dynamic consumer segmentation and prediction of an optimal tariff. Finally, comparative study between overlapping and non-overlapping windows and their impact on the choice of an optimal tariff was undertaken what revealed that significant improvements could be reported due to tariff changes. Specifically, the percentage improvements, on average, were as follows: Irish data—0.40–0.43%; Australian data—0.96–1.08%; and London data—0.68–0.93%. Assuming that the overall capacity of the system is approx. 45,000 MW in Poland, thus the improvements may deliver elasticity of electricity demand which is between 193.5 MW (0.43%) and 486 MW(1.08%). Those values are considered a significant from market balancing perspective. 

The direction for the future work will be to develop a fully scalable system (along with the results which are interpretable) for a large number of time series in the data stream, in the presence of:

- Concept drift of different kinds, such as incremental, recurring, sudden, or gradual;
- unstable number of sources (some sensors are newly created while other removed);
- heterogeneous and missing recordings;
- irregularly spaced data; and
- assuming application of other approaches for classifying incoming continuous data in dynamic systems e.g., stochastic learning weak estimators.

Due to that, we will investigate different incrementally computable time series similarity measures. In the future, we will investigate the influence (sensitivity of the algorithm) of the input parameters on the final results.

## Figures and Tables

**Table 1:** Matching between Fourier coefficient and electricity consumption behaviors.

Fourier Coefficients No.	Non Overlapping Windows	Overlapping Windows
1–6	20 days–120 days	14 days–84 days
7–30	4 days–17 days 3 h	2 days 19 h–12 days
31–240	12 h–3 days 21 h	8.4 h–2 days 17 h
>240	<12 h	<8.4 h

**Table 2:** Simulation of households’ electricity consumption characteristics based on different tariff group rates for non-overlapping windows.

	Min	1st Quartile	Median	Mean	3rd Quartile	Max
Best vs. worst individual tariff for each batch	2.39%	5.76%	7.67%	8.08%	9.88%	19.27%
Best individual tariff for each batch vs. best individual tariff for the entire period	0.00%	0.06%	0.21%	0.28%	0.40%	2.39%
Number of dynamic individual tariff change	0.00	2.00	5.00	4.81	7.00	12.00

**Table 3:** Simulation of households’ electricity consumption characteristics based on different tariff group rates for overlapping windows.

	Min	1st Quartile	Median	Mean	3rd Quartile	Max
Best vs. worst individual tariff for each batch	2.68%	6.27%	8.13%	8.47%	10.32%	19.28%
Best individual tariff for each batch vs. best individual tariff for the entire period	0.00%	0.23%	0.43%	0.51%	0.69%	3.52%
Number of dynamic individual tariff change	0.00	18.00	25.00	24.40	32.00	50.00

**Table 4:** Sample of the upper-triangular matrix of the ARI indexes obtained based on the ClipStream algorithm for non-overlapping windows.

	$B_{2}$	$B_{3}$	$B_{4}$	$B_{5}$	$B_{6}$	$B_{7}$	$B_{8}$	$B_{9}$	$B_{10}$
$B_{1}$	0.100	0.088	0.062	0.062	0.062	0.040	0.040	0.040	0.049
$B_{2}$		0.098	0.080	0.067	0.067	0.038	0.038	0.038	0.060
$B_{3}$			0.097	0.084	0.084	0.068	0.068	0.068	0.075
$B_{4}$				0.166	0.166	0.115	0.115	0.115	0.059
$B_{5}$					1	0.199	0.199	0.199	0.064
$B_{6}$						0.199	0.199	0.199	0.064
$B_{7}$							1	1	0.063
$B_{8}$								1	0.063
$B_{9}$									0.063

**Table 5:** Statistics of the ARI indexes for non-overlapping windows.

Clustering Algorithm	Min	1st Quartile	Median	Mean	3rd Quartile	Max
Extended TS-Stream (Fourier coeff.)	0.014	0.024	0.033	0.035	0.043	0.070
Extended TS-Stream (Fourier coeff., concept drift)	0.014	0.024	0.033	0.035	0.043	0.070
ClipStream (concept drift)	0.026	0.057	0.067	0.119	0.097	**1.000**
ClipStream (without concept drift)	0.021	0.054	0.070	0.079	0.091	0.232
ClipStream (Fourier coeff., concept drift)	0.029	0.053	0.066	0.120	0.082	**1.000**
ClipStream (Fourier coeff., without concept drift)	0.025	0.049	0.065	0.065	0.077	0.113
Histogram-based	**0.149**	**0.230**	**0.309**	**0.335**	**0.419**	0.740

**Table 6:** Statistics of the weighted volatility for non-overlapping windows.

Clustering Algorithm	Min	1st Quartile	Median	Mean	3rd Quartile	Max
Extended TS-Stream (Fourier coeff.)	**15.10**	**18.93**	**21.06**	**23.22**	**26.15**	37.18
Extended TS-Stream (Fourier coeff., concept drift)	**15.10**	**18.93**	**21.06**	**23.22**	**26.15**	37.18
ClipStream (concept drift)	20.50	27.40	32.69	37.08	47.93	61.93
ClipStream (without concept drift)	16.87	24.48	32.28	36.15	49.66	69.65
ClipStream (Fourier coeff., concept drift)	39.95	42.87	52.24	58.49	72.21	95.98
ClipStream (Fourier coeff., without concept drift)	39.95	42.87	52.24	55.83	64.88	90.06
Histogram-based	17.01	20.45	24.46	25.71	31.47	**36.05**

**Table 7:** Statistics of the tariffs improvement for non-overlapping windows.

Clustering Algorithm	Min	1st Quartile	Median	Mean	3rd Quartile	Max
Extended TS-Stream (Fourier coeff.)	**0.00%**	**0.10%**	**0.20%**	**0.40%**	0.50%	**1.80%**
Extended TS-Stream (Fourier coeff., concept drift)	**0.00%**	**0.10%**	**0.20%**	**0.40%**	0.50%	**1.80%**
ClipStream (concept drift)	−0.20%	**0.10%**	**0.20%**	0.30%	0.40%	1.50%
ClipStream (without concept drift)	−0.10%	**0.10%**	**0.20%**	0.30%	0.50%	1.50%
ClipStream (Fourier coeff., concept drift)	−0.10%	0.00%	0.10%	**0.40%**	**0.90%**	**1.80%**
ClipStream (Fourier coeff., without concept drift)	−0.10%	0.00%	0.10%	**0.40%**	0.80%	**1.80%**
Histogram-based	**0.00%**	**0.10%**	**0.20%**	**0.40%**	0.50%	**1.80%**

**Table 8:** Statistics of the predicted tariffs improvement comparing to the G11 for non-overlapping windows.

Clustering Algorithm	Min	1st Quartile	Median	Mean	3rd Quartile	Max
Extended TS-Stream (Fourier coeff.)	−8.89%	−0.56%	−0.09%	0.00%	0.45%	5.73%
Extended TS-Stream (Fourier coeff., concept drift)	−8.89%	-0.56%	−0.09%	0.00%	0.45%	5.73%
ClipStream (concept drift)	−3.17%	−0.34%	**−0.03%**	**0.31%**	**0.61%**	**8.46%**
ClipStream (without concept drift)	**−2.90%**	**−0.32%**	−0.04%	0.28%	0.53%	8.19%
ClipStream (Fourier coeff., concept drift)	−5.76%	−0.49%	−0.10%	−0.05%	0.40%	2.90%
ClipStream (Fourier coeff., without concept drift)	−5.76%	−0.49%	−0.10%	−0.05%	0.40%	3.25%
Histogram-based	−6.44%	−0.51%	−0.11%	−0.01%	0.41%	4.25%

**Table 9:** Summary results in terms of the average improvements on non-overlapping and overlapping windows.

Clustering Algorithm	Tariff Improvement		Tariffs Improvement Comparing to the G11	
	Non-Overlapping	Overlapping	Non-Overlapping	Overlapping
Extended TS-Stream (Fourier coeff.)	**0.40%**	**0.20%**	0.00%	0.21%
Extended TS-Stream (Fourier coeff., concept drift)	**0.40%**	0.10%	0.00%	0.20%
ClipStream (concept drift)	0.30%	0.10%	**0.31%**	**0.43%**
ClipStream (without concept drift)	0.30%	0.10%	0.28%	**0.43%**
ClipStream (Fourier coeff., concept drift)	**0.40%**	**0.20%**	−0.05%	0.14%
ClipStream (Fourier coeff., without concept drift)	**0.40%**	**0.20%**	−0.05%	0.15%
Histogram-based	**0.40%**	**0.20%**	−0.01%	0.16%

**Table 10:** Summary results in terms of the average improvements on non-overlapping and overlapping windows for Australian data.

Clustering Algorithm	Tariff Improvement		Tariffs Improvement Comparing to the G11	
	Non-Overlapping	Overlapping	Non-Overlapping	Overlapping
Extended TS-Stream (Fourier coeff.)	**0.96%**	0.14%	−0.18%	0.84%
Extended TS-Stream (Fourier coeff., concept drift)	**0.96%**	0.76%	−0.18%	0.84%
ClipStream (concept drift)	0.92%	**0.77%**	**0.19%**	**1.08%**
ClipStream (without concept drift)	0.91%	0.74%	0.18%	1.06%
ClipStream (Fourier coeff., concept drift)	0.93%	0.76%	−0.15%	0.82%
ClipStream (Fourier coeff., without concept drift)	0.93%	0.76%	−0.15%	0.83%
Histogram-based	0.91%	**0.77%**	−0.01%	0.16%

**Table 11:** Summary results in terms of the average improvements on non-overlapping and overlapping windows for London data.

Clustering Algorithm	Tariff Improvement		Tariffs Improvement Comparing to the G11	
	Non-Overlapping	Overlapping	Non-Overlapping	Overlapping
Extended TS-Stream (Fourier coeff.)	**0.93%**	0.10%	0.39%	0.59%
Extended TS-Stream (Fourier coeff., concept drift)	0.19%	0.10%	0.39%	0.57%
ClipStream (concept drift)	0.35%	**0.22%**	0.36%	0.62%
ClipStream (without concept drift)	0.39%	0.21%	0.39%	0.65%
ClipStream (Fourier coeff., concept drift)	0.26%	0.10%	0.23%	0.49%
ClipStream (Fourier coeff., without concept drift)	0.27%	0.10%	0.23%	0.50%
Histogram-based	0.15%	0.07%	**0.55%**	**0.68%**

## Appendix A. Results Based on Irish Data Set

**Table A1:** Statistics of the ARI indexes for overlapping windows.

Clustering Algorithm	Min	1st Quartile	Median	Mean	3rd Quartile	Max
Extended TS-Stream (Fourier coeff.)	0.003	0.019	0.025	0.031	0.033	0.264
Extended TS-Stream (Fourier coeff., concept drift)	0.003	0.024	0.034	0.044	0.050	0.326
ClipStream (concept drift)	0.011	0.046	0.059	0.091	0.082	**1.000**
ClipStream (without concept drift)	0.010	0.051	0.067	0.083	0.095	0.547
ClipStream (Fourier coeff., concept drift)	0.019	0.047	0.059	0.079	0.072	**1.000**
ClipStream (Fourier coeff., without concept drift)	0.020	0.049	0.062	0.070	0.076	0.405
Histogram-based	**0.222**	**0.348**	**0.467**	**0.486**	**0.597**	0.991

**Table A2:** Statistics of the tariffs improvement for overlapping windows.

Clustering Algorithm	Min	1st Quartile	Median	Mean	3rd Quartile	Max
Extended TS-Stream (Fourier coeff.)	−0.20%	**0.00%**	**0.10%**	**0.20%**	0.20%	0.80%
Extended TS-Stream (Fourier coeff., concept drift)	−0.20%	**0.00%**	**0.10%**	0.10%	0.20%	0.80%
ClipStream (concept drift)	−0.30%	**0.00%**	**0.10%**	0.10%	0.20%	0.80%
ClipStream (without concept drift)	−0.20%	**0.00%**	**0.10%**	0.10%	0.20%	0.90%
ClipStream (Fourier coeff., concept drift)	−0.20%	**0.00%**	**0.10%**	**0.20%**	**0.30%**	**1.00%**
ClipStream (Fourier coeff., without concept drift)	−0.20%	**0.00%**	**0.10%**	**0.20%**	**0.30%**	**1.00%**
Histogram-based	−0.20%	**0.00%**	**0.10%**	**0.20%**	**0.30%**	0.90%

**Table A3:** Statistics of the weighted volatility for overlapping windows.

Clustering Algorithm	Min	1st Quartile	Median	Mean	3rd Quartile	Max
Extended TS-Stream (Fourier coeff.)	15.94	22.27	25.83	26.28	29.97	38.21
Extended TS-Stream (Fourier coeff., concept drift)	**14.09**	20.76	25.22	25.09	28.67	39.20
ClipStream (concept drift)	19.15	24.61	28.41	29.57	32.50	59.14
ClipStream (without concept drift)	21.73	29.45	37.42	39.64	45.89	82.29
ClipStream (Fourier coeff., concept drift)	24.31	42.71	53.23	55.94	68.91	99.47
ClipStream (Fourier coeff., without concept drift)	32.69	45.22	53.85	58.30	69.60	106.51
Histogram-based	15.73	**19.59**	**23.45**	**24.29**	**27.19**	**36.41**

**Table A4:** Statistics of the predicted tariffs improvement comparing to the G11 for overlapping windows.

Clustering Algorithm	Min	1st Quartile	Median	Mean	3rd Quartile	Max
Extended TS-Stream (Fourier coeff.)	−9.28%	−0.40%	0.10%	0.21%	0.74%	5.45%
Extended TS-Stream (Fourier coeff., concept drift)	−8.52%	−0.40%	0.08%	0.20%	0.73%	6.22%
ClipStream (concept drift)	**−2.77%**	**−0.17%**	0.07%	**0.43%**	0.74%	**7.90%**
ClipStream (without concept drift)	−3.04%	**−0.17%**	0.08%	**0.43%**	**0.75%**	7.51%
ClipStream (Fourier coeff., concept drift)	−7.71%	−0.42%	0.11%	0.14%	0.68%	4.53%
ClipStream (Fourier coeff., without concept drift)	−7.58%	−0.42%	**0.12%**	0.15%	0.66%	4.69%
Histogram-based	−7.40%	−0.38%	0.08%	0.16%	0.65%	5.14%

**Table A5:** Statistics of the predicted tariffs improvement comparing to the random tariff for non-overlapping windows.

Clustering Algorithm	Min	1st Quartile	Median	Mean	3rd Quartile	Max
Extended TS-Stream (Fourier coeff.)	−6.28%	−0.10%	1.66%	2.50%	4.63%	13.45%
Extended TS-Stream (Fourier coeff., concept drift)	−6.28%	−0.10%	1.66%	2.50%	4.63%	13.45%
ClipStream (concept drift)	**−2.61%**	**0.00%**	**2.17%**	**2.80%**	**4.81%**	**14.35%**
ClipStream (without concept drift)	−3.69%	−0.01%	2.11%	2.77%	4.76%	**14.35%**
ClipStream (Fourier coeff., concept drift)	−6.53%	−0.12%	1.43%	2.45%	4.60%	13.27%
ClipStream (Fourier coeff., without concept drift)	−6.53%	−0.12%	1.43%	2.45%	4.60%	13.27%
Histogram-based	−6.47%	−0.10%	1.50%	2.49%	4.64%	13.45%

**Table A6:** Statistics of the predicted tariffs improvement comparing to the random tariff for overlapping windows.

Clustering Algorithm	Min	1st Quartile	Median	Mean	3rd Quartile	Max
Extended TS-Stream (Fourier coeff.)	−6.55%	0.13%	1.89%	2.69%	4.79%	13.48%
Extended TS-Stream (Fourier coeff., concept drift)	−6.63%	0.09%	1.82%	2.68%	4.80%	13.38%
ClipStream (concept drift)	−2.76%	0.12%	2.32%	**2.91%**	4.92%	**14.45%**
ClipStream (without concept drift)	**−2.51%**	0.13%	**2.34%**	**2.91%**	**4.96%**	14.40%
ClipStream (Fourier coeff., concept drift)	−6.53%	0.15%	1.66%	2.63%	4.76%	13.31%
ClipStream (Fourier coeff., without concept drift)	−6.16%	0.15%	1.70%	2.63%	4.74%	13.46%
Histogram-based	−6.64%	**0.17%**	1.81%	2.65%	4.75%	14.02%

## Appendix B. Results Based on Australian Data Set

**Table A7:** Simulation of households’ electricity consumption characteristics based on different tariff group rates for non-overlapping windows.

	Min	1st Quartile	Median	Mean	3rd Quartile	Max
Best vs worst individual tariff for each batch	2.86%	5.53%	6.99%	7.60%	8.96%	48.80%
Best individual tariff for each batch vs best individual tariff for the entire period	0.00%	0.66%	1.08%	1.04%	1.363%	4.26%
Number of dynamic individual tariff change	0.00	4.00	6.00	6.22	8.00	13.00

**Table A8:** Simulation of households’ electricity consumption characteristics based on different tariff group rates for overlapping windows.

	Min	1st Quartile	Median	Mean	3rd Quartile	Max
Best vs worst individual tariff for each batch	3.16%	6.60%	8.21%	8.79%	10.27%	49.45%
Best individual tariff for each batch vs best individual tariff for the entire period	0.00%	1.32%	1.70%	1.67%	1.95%	6.63%
Number of dynamic individual tariff change	0.00	16.00	24.00	22.97	30.00	50.00

**Table A9:** Statistics of the ARI indexes for non-overlapping windows.

Clustering Algorithm	Min	1st Quartile	Median	Mean	3rd Quartile	Max
Extended TS-Stream (Fourier coeff.)	0.024	0.047	0.063	0.066	0.082	0.165
Extended TS-Stream (Fourier coeff., concept drift)	0.024	0.047	0.063	0.066	0.082	0.165
ClipStream (concept drift)	0.001	0.040	0.058	0.078	0.101	**1.000**
ClipStream (without concept drift)	0.001	0.040	0.057	0.071	0.094	0.245
ClipStream (Fourier coeff., concept drift)	0.085	0.134	0.149	0.188	0.169	**1.000**
ClipStream (Fourier coeff., without concept drift)	0.085	0.127	0.149	0.154	0.171	0.300
Histogram-based	**0.286**	**0.400**	**0.457**	**0.491**	**0.524**	0.935

**Table A10:** Statistics of the tariffs improvement for non-overlapping windows.

Clustering Algorithm	Min	1st Quartile	Median	Mean	3rd Quartile	Max
Extended TS-Stream (Fourier coeff.)	**−0.06%**	0.03%	0.21%	**0.96%**	0.75%	**13.36%**
Extended TS-Stream (Fourier coeff., concept drift)	**−0.06%**	0.03%	0.21%	**0.96%**	0.75%	**13.36%**
ClipStream (concept drift)	−0.22%	0.05%	**0.27%**	0.92%	0.65%	12.13%
ClipStream (without concept drift)	−0.22%	0.04%	0.25%	0.91%	0.65%	12.13%
ClipStream (Fourier coeff., concept drift)	−0.13%	0.05%	0.20%	0.93%	0.74%	**13.36%**
ClipStream (Fourier coeff., without concept drift)	−0.13%	**0.06%**	0.23%	0.93%	0.74%	**13.36%**
Histogram-based	−0.11%	0.03%	0.21%	0.91%	**0.76%**	11.74%

**Table A11:** Statistics of the ARI indexes for overlapping windows.

Clustering Algorithm	Min	1st Quartile	Median	Mean	3rd Quartile	Max
Extended TS-Stream (Fourier coeff.)	0.015	0.037	0.049	0.067	0.079	0.438
Extended TS-Stream (Fourier coeff., concept drift)	−0.008	0.037	0.052	0.066	0.080	0.433
ClipStream (concept drift)	0.000	0.037	0.055	0.086	0.094	**1.000**
ClipStream (without concept drift)	−0.004	0.038	0.059	0.080	0.099	0.654
ClipStream (Fourier coeff., concept drift)	0.040	0.120	0.142	0.165	0.164	**1.000**
ClipStream (Fourier coeff., without concept drift)	0.040	0.120	0.142	0.152	0.167	0.515
Histogram-based	**0.218**	**0.405**	**0.537**	**0.540**	**0.648**	0.996

**Table A12:** Statistics of the tariffs improvement for overlapping windows.

Clustering Algorithm	Min	1st Quartile	Median	Mean	3rd Quartile	Max
Extended TS-Stream (Fourier coeff.)	−0.17%	**0.02%**	0.10%	0.14%	**0.23%**	**10.23%**
Extended TS-Stream (Fourier coeff., concept drift)	−0.19%	0.00%	0.04%	0.76%	0.15%	10.15%
ClipStream (concept drift)	−0.32%	0.00%	**0.11%**	**0.77%**	**0.23%**	**10.23%**
ClipStream (without concept drift)	−0.34%	−0.03%	0.07%	0.74%	0.21%	10.21%
ClipStream (Fourier coeff., concept drift)	−0.18%	0.00%	0.04%	0.76%	0.15%	10.15%
ClipStream (Fourier coeff., without concept drift)	−0.15%	0.00%	0.03%	0.76%	0.14%	10.14%
Histogram-based	**−0.13%**	0.01%	0.05%	**0.77%**	0.15%	10.15%

**Table A13:** Statistics of the weighted volatility for non-overlapping windows.

Clustering Algorithm	Min	1st Quartile	Median	Mean	3rd Quartile	Max
Extended TS-Stream (Fourier coeff.)	**4.33**	**5.79**	**7.70**	**7.65**	**9.13**	**10.89**
Extended TS-Stream (Fourier coeff., concept drift)	**4.33**	**5.79**	**7.70**	**7.65**	**9.13**	**10.89**
ClipStream (concept drift)	5.18	8.06	11.45	12.86	14.33	28.73
ClipStream (without concept drift)	5.18	8.06	12.03	12.9	14.33	28.73
ClipStream (Fourier coeff., concept drift)	12.29	19.83	24.04	23.94	27.91	42.01
ClipStream (Fourier coeff., without concept drift)	12.29	19.83	23.15	24.1	27.91	42.01
Histogram-based	5.19	6.73	8.72	8.64	10.29	11.87

**Table A14:** Statistics of the weighted volatility for overlapping windows.

Clustering Algorithm	Min	1st Quartile	Median	Mean	3rd Quartile	Max
Extended TS-Stream (Fourier coeff.)	**4.46**	**5.10**	**6.92**	**6.705**	8.05	**9.09**
Extended TS-Stream (Fourier coeff., concept drift)	4.66	5.27	6.94	13.21	**7.61**	70.76
ClipStream (concept drift)	7.88	12.95	16.15	19.38	23.38	37.46
ClipStream (without concept drift)	7.88	12.27	17.82	19.62	25.12	37.46
ClipStream (Fourier coeff., concept drift)	12.85	22.7	24.62	25.02	30.10	37.61
ClipStream (Fourier coeff., without concept drift)	11.94	19.18	24.62	23.63	28.04	33.60
Histogram-based	4.83	6.22	8.251	7.98	9.554	10.32

**Table A15:** Statistics of the predicted tariffs improvement comparing to the G11 for non-overlapping windows.

Clustering Algorithm	Min	1st Quartile	Median	Mean	3rd Quartile	Max
Extended TS-Stream (Fourier coeff.)	−6.29%	−1.12%	−0.27%	−0.18%	0.62%	14.57%
Extended TS-Stream (Fourier coeff., concept drift)	−6.29%	−1.12%	−0.27%	−0.18%	0.62%	14.57%
ClipStream (concept drift)	**−4.69%**	−0.79%	**−0.09%**	**0.19%**	**0.75%**	23.41%
ClipStream (without concept drift)	**−4.69%**	−0.76%	−0.10%	0.18%	0.73%	**24.80%**
ClipStream (Fourier coeff., concept drift)	−4.97%	−1.12%	−0.21%	−0.15%	0.64%	12.68%
ClipStream (Fourier coeff., without concept drift)	−5.39%	−1.07%	−0.23%	−0.15%	0.62%	13.92%
Histogram-based	−6.44%	**−0.51%**	−0.11%	−0.01%	0.41%	4.25%

**Table A16:** Statistics of the predicted tariffs improvement comparing to the G11 for overlapping windows.

Clustering Algorithm	Min	1st Quartile	Median	Mean	3rd Quartile	Max
Extended TS-Stream (Fourier coeff.)	−5.09%	−0.33%	**0.74%**	0.84%	1.77%	15.94%
Extended TS-Stream (Fourier coeff., concept drift)	−5.21%	−0.38%	0.73%	0.84%	1.81%	16.34%
ClipStream (concept drift)	−5.03%	0.08%	0.72%	**1.08%**	1.73%	23.12%
ClipStream (without concept drift)	**−5.00%**	**0.03%**	0.71%	1.06%	1.66%	**23.65%**
ClipStream (Fourier coeff., concept drift)	−6.33%	−0.44%	0.70%	0.82%	**1.82%**	16.83%
ClipStream (Fourier coeff., without concept drift)	−6.28%	−0.43%	0.71%	0.83%	**1.82%**	16.98%
Histogram-based	−7.40%	−0.38%	0.08%	0.16%	0.65%	5.14%

## Appendix C. Results Based on London Data Set

**Table A17:** Simulation of households’ electricity consumption characteristics based on different tariff group rates for non-overlapping windows.

	Min	1st Quartile	Median	Mean	3rd Quartile	Max
Best vs worst individual tariff for each batch	2.39%	4.79%	6.61%	7.21%	8.82%	33.37%
Best individual tariff for each batch vs best individual tariff for the entire period	0.00%	0.08%	0.24%	0.35%	0.51%	2.87%
Number of dynamic individual tariff change	0.00	3.00	5.00	4.90	7.00	14.00

**Table A18:** Simulation of households’ electricity consumption characteristics based on different tariff group rates for overlapping windows.

	Min	1st Quartile	Median	Mean	3rd Quartile	Max
Best vs worst individual tariff for each batch	2.72%	5.33%	7.22%	7.71%	9.43%	36.14%
Best individual tariff for each batch vs best individual tariff for the entire period	0.00%	0.26%	0.52%	0.62%	0.87%	3.36%
Number of dynamic individual tariff change	0.00	18.00	25.00	23.95	31.00	47.00

**Table A19:** Statistics of the ARI indexes for non-overlapping windows.

Clustering algorithm	Min	1st Quartile	Median	Mean	3rd Quartile	Max
Extended TS-Stream (Fourier coeff.)	0.000	0.066	0.081	0.080	0.100	0.199
Extended TS-Stream (Fourier coeff., concept drift)	0.000	0.066	0.081	0.080	0.100	0.199
ClipStream (concept drift)	0.080	0.140	0.173	0.213	0.215	**1.000**
ClipStream (without concept drift)	0.078	0.130	0.164	0.173	0.204	0.344
ClipStream (Fourier coeff., concept drift)	0.074	0.112	0.143	0.162	0.174	**1.000**
ClipStream (Fourier coeff., without concept drift)	−0.001	0.110	0.136	0.136	0.168	0.368
Histogram-based	**0.224**	**0.333**	**0.368**	**0.417**	**0.488**	0.889

**Table A20:** Statistics of the tariffs improvement for non-overlapping windows.

Clustering Algorithm	Min	1st Quartile	Median	Mean	3rd Quartile	Max
Extended TS-Stream (Fourier coeff.)	−0.13%	0.06%	**0.23%**	**0.93%**	**0.74%**	1.74%
Extended TS-Stream (Fourier coeff., concept drift)	−0.25%	0.01%	0.07%	0.19%	0.20%	1.72%
ClipStream (concept drift)	**−0.05%**	0.08%	0.20%	0.35%	0.41%	1.58%
ClipStream (without concept drift)	−0.05%	**0.11%**	0.22%	0.39%	0.47%	1.71%
ClipStream (Fourier coeff., concept drift)	−0.20%	0.01%	0.13%	0.26%	0.22%	**1.92%**
ClipStream (Fourier coeff., without concept drift)	−0.16%	0.02%	0.13%	0.27%	0.22%	**1.92%**
Histogram-based	−0.09%	0.03%	0.09%	0.15%	0.27%	0.72%

**Table A21:** Statistics of the ARI indexes for overlapping windows.

Clustering Algorithm	Min	1st Quartile	Median	Mean	3rd Quartile	Max
Extended TS-Stream (Fourier coeff.)	0.036	0.064	0.077	0.090	0.099	0.442
Extended TS-Stream (Fourier coeff., concept drift)	0.031	0.064	0.078	0.090	0.098	0.447
ClipStream (concept drift)	0.060	0.125	0.150	0.176	0.190	**1.000**
ClipStream (without concept drift)	0.060	0.121	0.149	0.168	0.191	0.744
ClipStream (Fourier coeff., concept drift)	0.053	0.111	0.143	0.164	0.183	**1.000**
ClipStream (Fourier coeff., without concept drift)	0.055	0.110	0.139	0.155	0.177	0.658
Histogram-based	**0.209**	**0.321**	**0.395**	**0.426**	**0.504**	0.984

**Table A22:** Statistics of the tariffs improvement for overlapping windows.

Clustering Algorithm	Min	1st Quartile	Median	Mean	3rd Quartile	Max
Extended TS-Stream (Fourier coeff.)	−0.21%	**0.02%**	0.08%	0.10%	0.16%	1.39%
Extended TS-Stream (Fourier coeff., concept drift)	−0.21%	0.00%	0.06%	0.10%	0.16%	1.39%
ClipStream (concept drift)	−0.37%	**0.02%**	**0.17%**	**0.22%**	0.34%	2.13%
ClipStream (without concept drift)	−0.41%	0.01%	**0.17%**	0.21%	**0.35%**	2.13%
ClipStream (Fourier coeff., concept drift)	−0.55%	−0.03%	0.04%	0.10%	0.15%	**2.43%**
ClipStream (Fourier coeff., without concept drift)	−0.38%	−0.03%	0.04%	0.10%	0.15%	**2.43%**
Histogram-based	**−0.17%**	0.00%	0.04%	0.07%	0.11%	1.14%

**Table A23:** Statistics of the weighted volatility for non-overlapping windows.

Clustering Algorithm	Min	1st Quartile	Median	Mean	3rd Quartile	Max
Extended TS-Stream (Fourier coeff.)	**6.75**	**8.47**	**9.84**	**10.11**	**12.16**	164.98
Extended TS-Stream (Fourier coeff., concept drift)	**6.75**	**8.47**	**9.84**	**10.11**	**12.16**	164.98
ClipStream (concept drift)	19.36	23.72	26.63	29.58	33.91	48.24
ClipStream (without concept drift)	19.36	24.10	26.48	29.81	37.19	48.24
ClipStream (Fourier coeff., concept drift)	27.91	43.52	48.81	51.61	63.26	85.77
ClipStream (Fourier coeff., without concept drift)	27.91	43.52	48.81	57.82	70.38	164.32
Histogram-based	9.36	10.37	11.50	12.46	14.73	**18.77**

**Table A24:** Statistics of the weighted volatility for overlapping windows.

Clustering Algorithm	Min	1st Quartile	Median	Mean	3rd Quartile	Max
Extended TS-Stream (Fourier coeff.)	**7.03**	**8.94**	**9.46**	**10.54**	**11.14**	**17.08**
Extended TS-Stream (Fourier coeff., concept drift)	**7.03**	**8.94**	**9.46**	**10.54**	**11.14**	**17.08**
ClipStream (concept drift)	16.79	20.30	23.10	25.41	29.59	43.31
ClipStream (without concept drift)	16.79	20.30	23.10	25.15	29.59	39.00
ClipStream (Fourier coeff., concept drift)	24.99	28.92	41.69	42.91	53.07	62.66
ClipStream (Fourier coeff., without concept drift)	24.99	28.92	41.69	42.33	51.61	62.66
Histogram-based	8.86	10.91	11.77	12.76	13.55	21.39

**Table A25:** Statistics of the predicted tariffs improvement comparing to the G11 for non-overlapping windows.

Clustering Algorithm	Min	1st Quartile	Median	Mean	3rd Quartile	Max
Extended TS-Stream (Fourier coeff.)	−5.37%	−0.67%	0.27%	0.39%	1.27%	7.14%
Extended TS-Stream (Fourier coeff., concept drift)	−5.37%	−0.67%	0.27%	0.39%	1.27%	7.14%
ClipStream (concept drift)	**−3.96%**	**−0.46%**	0.11%	0.36%	0.78%	12.72%
ClipStream (without concept drift)	**−3.96%**	−0.48%	0.10%	0.39%	0.79%	**13.81%**
ClipStream (Fourier coeff., concept drift)	−6.62%	−0.76%	0.18%	0.23%	1.08%	7.89%
ClipStream (Fourier coeff., without concept drift)	−6.62%	−0.75%	0.18%	0.23%	1.10%	7.89%
Histogram-based	−6.43%	−0.68%	**0.31%**	**0.55%**	**1.52%**	10.97%

**Table A26:** Statistics of the predicted tariffs improvement comparing to the G11 for overlapping windows.

Clustering Algorithm	Min	1st Quartile	Median	Mean	3rd Quartile	Max
Extended TS-Stream (Fourier coeff.)	−7.41%	−0.53%	0.41%	0.59%	1.53%	8.66%
Extended TS-Stream (Fourier coeff., concept drift)	−5.83%	−0.50%	**0.42%**	0.57%	1.51%	7.66%
ClipStream (concept drift)	−3.95%	**−0.32%**	0.28%	0.62%	1.10%	13.26%
ClipStream (without concept drift)	**−5.14%**	**−0.32%**	0.31%	0.65%	1.22%	13.27%
ClipStream (Fourier coeff., concept drift)	−6.08%	−0.51%	0.37%	0.49%	1.33%	7.86%
ClipStream (Fourier coeff., without concept drift)	−6.71%	−0.48%	0.37%	0.50%	1.37%	8.43%
Histogram-based	−7.61%	−0.48%	0.37%	**0.68%**	**1.64%**	**10.90%**
